# Relationship of Exclusion From Physical Education and Bullying in Students With Specific Developmental Disorder of Scholastic Skills

**DOI:** 10.3389/ijph.2022.1604161

**Published:** 2022-08-24

**Authors:** Ondrej Jesina, Ladislav Baloun, Martin Kudlacek, Aneta Dolezalova, Petr Badura

**Affiliations:** Faculty of Physical Culture, Palacký University, Olomouc, Olomouc, Czechia

**Keywords:** adapted physical activity, elementary school, special education needs, specific learning disability, bullying at school

## Abstract

**Objectives:** This study aimed to analyze the relationships among exclusion from PE, gender, and bullying in adolescents with specific developmental disorder of scholastic skills (SDDSS) aged 11, 13, and 15 years in Czechia.

**Methods:** In total, the final research sample consisted of 13,953 students (49.4% boys) from the 2013/2014 Health Behaviour in School-aged Children survey. Chi-square tests and regression models stratified by presence of SDDSS diagnosis were used to assess the relationships between non-involvement in PA and bullying.

**Results:** Students diagnosed with SDDSS (12.4% of the sample) were more likely to be excluded from physical education (PE) than students without this diagnosis. This exclusion was associated with higher odds of bullying victimization and perpetration. Our findings further showed that male gender plays a significant role for bullying perpetration for both groups (with and without SDDSS) investigated in the present study.

**Conclusion:** Higher likelihood of aggressive behavior occurs in students who are excluded from PE, including students with SDDSS.

## Introduction

Physical activity (PA) plays a critical role in the social inclusion and the overall mental, physical, social and spiritual development of students with special educational needs (SEN) including specific developmental disorder of scholastic skills (SDDSS). We used diagnosis terminology of World Health Organization from publication International statistical classification of diseases and related health problems 10th revision (5th eds.). For the purpose of this study the acronym SDDSS priority means code F81 Specific developmental disorder of scholastic skills however often is combined with F82 Specific developmental disorder of motor function. This connection is categorized like F83 Mixed specific developmental disorders. It is important to emphasize that these three categories are often combined with other diagnoses such as Attention Deficit Hyperactivity Disorder (ADHD). Many authors deal with the topic of inclusive physical education (PE) and participation of students with SEN [1–3]. Pontifex et al. [4] also mention the positive role of PA in reducing barriers to learning in children with SDDSS. For students with SDDSS, this means that if they do not encounter a diverse range of PAs and have limited social contact enabling them to share emotions, they are likely to have restricted access to PA later in life with negative impact on their own health and quality of life [5]. School-based PE provides a suitable room for practicing PA and inclusion of students with SDDSS as it is a compulsory part of education in most European countries [6]. Students with SDDSS (especially in combination with other SEN) are often excluded from PE in elementary and high schools without proper reasons [7]. This process of exclusion (non-participation) is possible not only by the Czech legislation, but especially by the approach of school administration because school headmaster/headmistress represent authority administering the exclusion process [8]. The school headmaster/headmistress are responsible for assessing the justification and the subsequent consent to the release of students from physical education. However, in the vast majority of cases, he does not question the statements of the registering doctors and agrees without comment with the overall release or with significant content adjustments. These possible adjustments then *de facto* mean the same as formal release overall [5].

### Basic Description of Specific Developmental Disorder of Scholastic Skills

In the concept of special education SDDSS as specific learning disabilities can be defined as an unexpected and unexplained condition that can affect a child with average or above average intellect. It is described by a significant delay in one or more areas of learning [9]. This is mainly the area of perceptual and motor deficiencies, which extend into other subcategories f. e. dysgraphia, dyscalculia, dyslexia [10, 11]. A number of studies [12–15] show frequent comorbidity of SDDSS with Attention Deficit Hyperactivity Disorder (ADHD). Lack of exercise is reported to engender psychological imbalance, deficit in the formation of social relationships, or aggression [16]. Aggression in children with SDDSS may be caused by uncertainty, fatigue, fear, and subsequent disappointment from constant failure. Aggression related to its social environment can reach the dimensions of bullying.

### Aggression and Bullying

There may be several reasons why children with SDDSS bully or or they are bullied by their classmates. They may want to attract attention or react aggressively because they are excluded from their peers [17]. Multiple authors agree on the definition of bullying and perceive it as a systematic abuse of power, which is manifested in three basic criteria: 1) deliberate, respectively, aggressive behavior towards another, 2) repetition, and 3) imbalance of power between the victim and aggressor [18–20]. Bullying among children and adolescents is a global public health issue as it is associated with negative childhood development [17, 21, 22]. Bullying of adolescents has a lasting impact on mental and physical health in a lifelong context [22, 23].

Gender differences in bullying in children and adolescents has been the subject of many studies with various results. The evidence [24–27] consistently reports that boys are more likely involved in bullying than girls. However, there are some studies [28, 29], showing girls being involved in bullying at higher rates or having broader experience with specific forms of bullying (cyberbullying and combined bullying) than boys. At the same time [30, 31], it was observed that there are no gender differences in specific groups, such as students with autism spectrum disorder, in bullying perpetration. However, female gender was a significant predictor of bullying victimization by students with SDDSS [32]. It is clear that in an inclusive education process, all students with differences, more specifically with SEN (including SDDSS) are potentially bullied.

### The Role of Physical Activities

Practice of PA (non-competitive) appears to be an excellent means for the transmission of values and helps promote prosocial attitudes [33] so it can be helpful in the prevention and treatment of bullying and decrease a risk of developing aggressive behaviors [34]. Mendez et al. [35] draw attention to the socially negative impacts in relation to the bullying of regular athletes and, conversely, draw attention to the positive effect in the case of students involved in educational and non-competitive physical activities. This can be fulfilled in a suitable way, especially by involvement in school physical education. A clear connection between students’ non-involvement in physical activities and a higher prevalence of bullying is documented by a number of previous findings by García-Hermoso et al [36]. However Fisher and Dzikus describe, when athletes bully each other, it appears that they are influenced by gender norms and significant others (e.g., peers, coaches) [37]. Kowalski [38] describes how the values of Western culture associated with sports activities, such as winning at all costs, using power and dominance to control others, and using the hierarchical structure of authorities, can contribute to bullying. The author considers the potential influence of the coach to be a central authority in the development of athletes. This can affect whether students are bullied to achieve sports-related goals. In the school context, the influence of the school PE teacher can therefore be considered.

### Thematic Context and Development Trends in the Czech Republic

Data shows reducing trends of bullying among children and adolescents at the Czech schools in recent decades [39], yet bullying rates in Czechia are still around average in comparison to countries from Organization for Economic Co-operation and Development (OECD) [40]. The OECD’s report that children and students with low academic performance (including those with SDDSS) are more likely to become victims of bullying. However, data on the relationship among PA, SDDSS and bullying is rare or missing. This is partly because the SDDSS is not considered a reason for being excluded from the compulsory school PE in most countries of the world. Due to the overuse of the process of exclusion from PE in the Czech Republic, especially in students with SDDSS, it is possible to fill this research gap. Based on previously established assumptions and findings about the relationship between bullying and SEN (including SDDSS) [26, 41–43] we assumed that children suffering from SDDSS might be at higher risk of being involved in bullying (either as a perpetrator or a victim), which could be further pronounced in case of their non-attendance to PE. The purpose of this study was to analyze the relationship among exclusion from PE, gender, and bullying in adolescents with SDDSS aged 11–15 years.

## Methods

The present study is based on an international study backed by the World Health Organization (WHO) titled Health Behaviour in School-aged Children (HBSC), which is the international collaborative research study on the lifestyle of schoolchildren. For nearly 40 years, HBSC has been providing cross-national data on health, well-being, social environment and health-related behaviors in the population of 11-year-olds, 13-year-olds and 15-year-olds. In each of the member countries, the cross-sectional data collection is carried out in 4-year intervals. The present study uses the data from the survey conducted in Czechia in the spring 2014.

### Sample

The 2013/2014 Czech sample was designed to comply with the requirements of the HBSC International Protocol [44]. To ensure national representativeness, it was stratified by region and type of school (ratio of primary schools to multi-year grammar schools). From the database of the Ministry of Education, Youth and Sports of the Czech Republic, 225 primary schools and 18 multi-year grammar schools were randomly selected. Out of 243 schools that were addressed only one refused to participate and was replaced by another school in its vicinity. In total, 16,298 students were enrolled in the schools that consented to participate. In each school, one class from the 5th, 7th, and 9th grades was chosen at random if there were two or more classes in the respective grades re. Overall, 14,569 students were present during the paper-and-pencil questionnaire survey, of which 30 refused to participate in the data collection (response rate at the individual level = 89.2%). Based on optical control, too many unanswered questions and missing data on age, gender or SDDSS diagnosis 206 questionnaires were removed from the sample.

In total, the final research sample consisted of 13,953 students. Of these, 1,737 students (1,123 boys and 614 girls) reported to be diagnosed with SDDSS. The students declared the diagnosis themselves, but with regard to the ratio of girls and boys, and was homogeneously distributed across grades. The frequency of occurrence of SDDSS is not easy to express. In Czechia, up to 10% corresponds to a qualified estimate with a data collection period [45]. The official source of the National Institute for Education, Education Counselling Centre and Centre for Continuing Education of Teachers established by the Ministry of Education, Youth and Sports of the Czech Republic speaks of 5–15% of students with SDDSS [46]. The same source also explains that the range is not accurate because they are in this group of students including students with varying severity of manifestations of the varied spectrum of SDDSS. At the same time, a further increase in the number of students is expected based on the refinement diagnostic procedures.

In this context, however, there is talk of students with more severe forms of SDDSS. The basic characteristics of the sample are presented in Table 1. The participation in the study was voluntary and anonymous. No incentives were offered to the respondents for their participation. The consent to carry out the study was obtained through the school headmaster/headmistress. The participants (or their parents/guardians) could opt out from the study at any moment or skip questions that made them feel uncomfortable. The study was approved by the authors’ institutional ethics committee under reg. no. 17/2013.

**TABLE 1:** Demographic characteristics of the sample: Health Behaviour in School-aged Children (Olomouc, Czechia. 2014).

	Boys	Girls	Total	Age (years)	
	n (%)	n (%)	n	M	SD
5th grade	2180 (49.1%)	2257 (50.9%)	4437	11.41	0.41
7th grade	2367 (49.4%)	2428 (50.6%)	4795	13.42	0.42
9th grade	2346 (49.7%)	2375 (50.3%)	4721	15.42	0.41
Total	6893 (49.4%)	7060 (50.6%)	13953		

% represents the relative rate of participants per row, M, mean; SD, standard deviation.

### Measures

Students indicated whether they have been diagnosed with eight specific long-term conditions, with one of them being *learning disability (dyslexia, dysgraphia, dysorthographia, dyscalculia).* The respondents who reported that they were diagnosed with learning disabilities were classified as suffering from SDDSS. This question has long been included in the pool of optional packages that can be picked up by the HBSC member countries who express their interest. For example, Finland [47] investigates the topic regularly in connection with other determinants examined.

*Bullying* was investigated using two questions adapted from the Olweus Bully/Victim Questionnaire [48] allowing to assess both *victimization* and *perpetration*. Those who reported that they have been bullied or have taken part in bullying another student(s) in the past couple of months at least once or twice were considered bullying victims and perpetrators, respectively, in subsequent analyses in line with a previous study using the Czech HBSC data [39]. The questions were preceded by the text explaining the term of bullying to respondents. In a systematic review of 27 instruments measuring the youth bullying experience, the Olweus questionnaire showed the strongest support for its psychometric properties [49]. Three forms of PA representing potentially protective factors against bullying were: 1) *participation in general PE*, where the respondents indicated whether they have been excluded/excused from PE (either to full extent or on a partial basis); 2) *participation in organized sport* (team and/or individual), with simple binary response options *yes* or *no* retrieved from a 6-item scale on organized activity participation showing adequate reliability for the population studies of an epidemiological nature [50]; 3) *moderate-to-vigorous physical activity* measuring number of days the respondents were physically active for at least 60 min [51], which was recommended as a brief surveillance measure by Biddle and others [52]. In line with the generally recognized recommendation for this age category (60 min every day), the item was dichotomized as 7 days vs. less often. As the study aimed at assessing the relationships among SDDSS, exclusion from PE and bullying, the latter two forms of PA represented control variables in the analyses.

Last, we used a *classmate support* scale–a measure with satisfactory convergent validity and test-retest indices, which have been used in the HBSC surveys since 1993/94 [53]. It consists of three items: 1) *The students in my class(es) enjoy being together*; 2) *Most of the students in my class(es) are friendly*; 3) *The students in my class accept me as I am*. Responses were rated on a 5-point Likert scale from *strongly disagree* to *strongly agree* and the overall score computed and treated as a continuous variable, with higher score indicating greater level of perceived classmate support. We used it as a control variable in the regression analyses because we anticipated that bullying is less likely to occur in classes with higher perceived support and vice versa.

### Statistical Analyses

All the analyses were carried out using the IBM SPSS 22 software (IBM Corp. Released 2013. IBM SPSS Statistics for Windows, Version 22.0. Armonk, NY: IBM Corp.). First, we described the composition of the sample with a focus on the rate of respondents diagnosed with SDDSS. Then, we compared the SDDSS and non-SDDSS with respect to the bullying status and participation or exclusion from PE using chi-square tests and the strength of the associations was estimated using Phi, with values of 0.1, 0.3, and 0.5 considered a small, medium, and large effect, respectively.

Next, we ran a series of multiple logistic regression analyses to assess the associations of exclusion from PE, gender and bullying status–separately for victimization and perpetration. To meet the aim of the study, the results of analyses are presented after being stratified by presence of SDDSS diagnosis. The regression analyses were adjusted for grade, perceived classmate support, as well as organized sport participation and overall level of PA as a suitable space to ventilate aggression. Statistical significance of all analyses was set to *α* = 0.05.

## Results

Regarding the participation in PE, 18.3% of the students with SDDSS were excluded from PE compared with 14.3% among the students without SDDSS. In other words, the students with SDDSS were excluded at a significantly higher rate (*p* < 0.001; Phi = 0.037) than their peers without SDDSS; however, the effect size was very small. The results show that in students with SDDSS, 23.5% have become victims of bullying at least once in recent months, compared to 16.5% observed in those not diagnosed with SDDSS (*p* < 0.001; Phi = 0.062).

Next, we observed that 28.3% of students with SDDSS, who were excluded from PE became victims of bullying, which is a rate higher by 6.0 p.p. (*p* = 0.026, Phi = 0.055) compared to 22.3% of students with SDDSS participating in PE. This implies that students with SDDSS who were excluded from PE were more often victims of bullying. Similar results were found regarding bullying perpetration. Out of the total number of students excluded from PE, 24.8% were involved in bullying other students on at least one occasion in the past couple of months. Among students participating in PE, 18.9% were involved in bullying of another person, which was significantly less than in those excluded from PE (*p* = 0.020; Phi = 0.057). With a dichotomous view of bullying from a perpetration perspective, the results show (*p* < 0.001; Phi = 0.056) that there were more bullying perpetrators with SDDSS (19.9%) than those without SDDSS (13.9%). The overall results show a higher prevalence of students with SDDSS (24.8%) excluded from PE who were involved in bullying of another person, compared to 15.7% without SDDSS (*p* < 0.001; Phi = 0.087).

The results (see Table 2) of the multiple regression analyses adjusted for grade, perceived classmate support, as well as other forms of PA, showed that gender [odds ratio (OR) = 0.98, 95% confidence interval (CI) = 0.76–1.26] did not play a significant role in students with SDDSS in terms of whether they were being bullied or not. This differed from the population without SDDSS, where boys were significantly more likely to become victims of bullying (OR = 1.19, 95% CI = 1.07–1.32). There were also higher odds of becoming a victim of bullying at least once in recent months when students were excluded from PE, even after accounting for overall PA and participation in organized sports. These findings applied to both students with SDDSS (OR = 1.40, 95% CI = 1.04–1.88) and students without SDDSS (OR = 1.25; 95% CI = 1.09–1.44).

**TABLE 2:** Multiple logistic regression: Odds ratios and 95% confidence intervals for bullying victimization stratified by presence or absence of diagnosis (Olomouc, Czechia. 2014).

	SDDSS diagnosed (*n* = 1601)		No SDDSS (*n* = 11455)	
	OR (95% CI)	p-value	OR (95% CI)	p-value
Gender (boy vs. girl)	0.98 (0.76–1.26)	0.888	**1.19 (1.07–1.32)**	**0.001**
Moderate-to-vigorous physical activity (60 min daily)	0.91 (0.67–1.22)	0.535	0.91 (0.80–1.03)	0.135
Participation in organised sport (yes)	1.03 (0.80–1.33)	0.811	1.01 (0.90–1.12)	0.917
Exclusion from PE (full or partial)	**1.40 (1.04–1.88)**	**0.028**	**1.25 (1.09–1.44)**	**0.001**

SDDSS, specific developmental disorder; PE, physical education; OR, odds ratio; CI, confidence interval.

The present model was adjusted for grade and perceived classmate support. Statistically significant ORs are indicated in bold (p < 0.05).

Boys both with and without SDDSS were more likely to act as bullying perpetrators on one or more occasion during the recent months (Table 3). Similar to victimization, exclusion from PE was associated with higher odds of bullying other students significantly. For students with SDDSS diagnosis, OR equaled 1.46 (95% CI = 1.07–1.99), and for those without SDDSS, OR was 1.25 (95% CI = 1.08–1.45).

**TABLE 3:** Multiple logistic regression: Odds ratios and 95% confidence intervals for bullying perpetration stratified by presence or absence of diagnosis (Olomouc, Czechia. 2014).

	SDDSS diagnosed (*n* = 1597)		No SDDSS (*n* = 11 442)	
	OR (95% CI)	p-value	OR (95% CI)	p-value
Gender (boy vs. girl)	**1.80 (1.36–2.39)**	**<0.001**	**2.07 (1.85–2.31)**	**<0.001**
Moderate-to-vigorous physical activity (60 min daily)	1.04 (0.77–1.41)	0.787	0.97 (0.85–1.11)	0.966
Participation in organised sport (yes)	0.95 (0.73–1.23)	0.674	0.89 (0.79–1.00)	0.051
Exclusion from PE (full or partial)	**1.46 (1.07–1.99)**	**0.016**	**1.25 (1.08–1.45)**	**0.003**

SDDSS, specific developmental disorder; PE, physical education; OR, odds ratio; CI, confidence interval.

The present model was adjusted for grade and perceived classmate support. Statistically significant ORs are indicated in bold (p < 0.05).

## Discussion

The purpose of this study was to analyze the relationship among various domains of PA (exclusion from PE, participation in organized sport, and level of moderate-to-vigorous PA), gender, and bullying in adolescents with SDDSS aged 11–15 years.

Our findings further show that students with SDDSS are slightly more likely to be excluded from PE than students without SDDSS (18.3% vs. 14.3%). Students with SDDSS who were excluded from PE also became victims of bullying and bullying perpetrators at higher rates in comparison with students with SDDSS who participated in PE. Based on these results, it appears that a higher likelihood of aggressive behavior occurs in students who are excluded from PE, including students with SDDSS. Students with SDDSS, who are excluded from PE therefore could be at even higher risk of being involved in bullying than students without SDDSS. Overall, this is a worrying fact given that mere presence of SDDSS symptoms does not establish a legal basis for exclusion from PE. We are not able to describe the reasons for release from PE, yet according to Czech legislative standards, this release must be carried out on the basis of health limits, not on the basis of our own request, which is not justified. For example, fear of social contact or physical activity is currently not acceptable according to applicable legislative standards.

Male gender was related to bullying perpetration for both groups examined in this study (both students with and without SDDSS). In case of bullying victimization, male gender was shown to be significant only for students without SDDSS. Our results support previous findings [24–27, 54] that boys are more likely to be involved in bullying than girls, but our results are in contrast with findings of Rose et al. [32] where female gender was a predictor of bullying victimization by students with SDDSS. Gender was identified as the strongest predictor for both groups of students (with and without SDDSS) in bullying perpetration. Male gender was not established as a predictor only in bullying victimization by SDDSS diagnosed students.

The results of a previous HBSC study [55] from 2010 showed the upward trend of students excluded from PE in Czechia, which is co-responsible for the global problem in the decrease of PA level in youth. For this reason, the Czech HBSC study also investigates participation in general PE and PA outside school and school facilities. The results show that about 8% of Czech students were excluded from compulsory PE, with the number of boys being higher than girls [55]. We can see a clear increasing tendency to avoid participating in PE, with 14.2% of students without SEN and 18.4% of students with SDDSS being excluded from PE in 2013/2014. In line with our results, Rose, Monda-Amaya and Espelage [43] state that students with SDDSS were 2–3 times more likely to be victimized than classmates without disabilities.

Sentenac et al. [56] investigated students with long-term illness from eleven countries examining the relationship between bullying and chronic illnesses in the HBSC study. They found that these children are more often victims of bullying (at least two or three times a month) than the other students. Those who declared some form of chronic illnesses were more likely to be victims of bullying. The study also showed low self-esteem, life dissatisfaction, and many health-related problems in students who have previously experienced bullying. Swedish self-reported cross-sectional study [57] discovered similar findings, that students with differences (including students with SDDSS) are more exposed to bullying.

According to the Czech legal standards (Education Act 561/2004), the school is obliged to supervise the health of students and prevent the occurrence of socially pathological behavior. In connection with this, it is necessary to mention that the school is often the initiator of the exclusion from PE or at least does not use its authority to prevent this exclusion, because according to the Education Act, the school (through the headmaster) is the entity that enables the excuse from PE.

Systematic exclusion (non-participation) increases the risk of bullying and creates conditions for the emergence of other non-desirable social behaviors. This is because students with SDDSS are involved in bullying more than students without SDDSS (whether as victims or aggressors). Also this “risk” is further exacerbated if they do not participate in PE (other types of PA did not show significant effects on bullying behavior) even after accounting for class climate effects. The classroom environment can often prevent bullying at the very beginning of this socially pathological behavior. Non-participation in PE also increases this “risk” of bullying among students without SDDSS. However, due to the absence of data on other possible limiting diagnoses (comorbidities), it is possible that the association of bullying with exclusion from PE can be explained only partially. It is PE that represents a potential environment for experiencing success, for relaxation and for creating positive social links and relationships.

Participation in PA can be an effective means of reducing the impact of bullying in relation to the psycho-social dimension of health [28]. The emotional states of the victims are full of anxiety, fear, and worries about where and when the next attack occurs. This primarily affects their study, rather than focusing on learning, focusing on finding ways to protect themselves from aggressors. The consequence is absence from school activities and places where there is a higher chance of being bullied. Usually the bullying victims are very lonely and admit having no close friends in the classroom [58]. Conflicts with family members and others around the victim may also be the result of bullying. Often, when victims encounter loneliness and social isolation from their classmates, they also show frustration at home. One form of expressing their irritation is to develop problematic relationships with their parents who are unaware of the fact that their child is a victim of bullying. The fragile relationship between parent and child further isolates victims because they not only lack social interaction at school, but find no support at home [59–61].

### Strengths and Limitations

The crucial strength of this study is its large and representative sample. Furthermore, It was based on the well-established and recognized HBSC study, with a strong methodological background regarding data collection procedures and construction of the questionnaire. There are some limitations of this study that must be noted. First, the obtained data was self-reported. Participants’ responses about their experience of bullying are subjective, the same situation is in the area of question asking whether a respondent has been diagnosed with learning disability (dyslexia, dysgraphia, dysorthographia, dyscalculia), which might be more prone to be biased. To address this limitation, future research should focus on mixed methods (with both students and school staff), and thus supplement the quantitative results with qualitative findings. Also in the present study, we did not possess data on the severity of the SDDSS, which could have had an effect on the associations observed with exclusion from PE or involvement in bullying. Second, measures used in the present study did not explicitly refer to cyberbullying. Consequently, the prevalence rates reflect only traditional forms of bullying and not cyberbullying. Third, the cross-sectional nature of the survey does not allow inference about causal relationships among the variables. Hence, another variable could explain both lack of PE participation and dependent variables (e.g., health limits could drive the bullying rather than the participation in PE).

### Conclusion

Students with SDDSS are more likely to be excluded from PE than students without this diagnosis. Concurrently, students with SDDSS who were excluded from PE also reported to be victims of bullying and involved in bullying perpetration at higher rates in comparison with students with SDDSS who participated in PE. Male gender was associated with bullying perpetration for both groups (students with and without SDDSS), whereas male gender was associated with bullying victimization only for students without SDDSS.

The results of this study could be used for the subsequent intervention focusing on the support of physical activities connected with the strengthening of relationships in the group of students within the teaching of PE. Findings can be interpreted with regard to potential adjustments of the inclusive education system in Czechia. Future research should concentrate on revealing causal pathways from exclusion from PE to engagement in bullying and identifying reasons for excessive exclusions from PE in students diagnosed with SDDSS.

The abolition of procedures leading to release from PE at the basis of diagnosis should be an integral part of the whole education system. Application of a system of support measures for students with SDDSS leading to health promotion is a necessity within inclusive approaches. The potential negative effects of ‘unnecessary’ release from PE on the psycho-social dimension of health can be far more extensive than, unfortunately, the professional pedagogical, as well as the medical community is often aware of.
